# Building a Narrative of Equity: Weaving Indigenous Approaches into Community-Engaged Research

**DOI:** 10.3390/ijerph17145148

**Published:** 2020-07-16

**Authors:** Lisa J. Hardy, Kevin Shaw, Amy Hughes, Elizabeth Hulen, Priscilla R. Sanderson, Candi Corrales, Travis Pinn, Jamie Esplain, R. Cruz Begay

**Affiliations:** 1 Department of Anthropology, Northern Arizona University, Flagstaff, AZ 86011, USA; travis.pinn@nau.edu; 2 Social Science Community Engagement Lab, Northern Arizona University, Flagstaff, AZ 86011, USA; 3 Center for Research and Evaluation on Education and Human Services, Montclair State University, Montclair, NJ 07043, USA; shawk@montclair.edu; 4 Cline Library, Northern Arizona University, Flagstaff, AZ 86011, USA; amy.hughes@nau.edu; 5 Department of Sociology, Portland State University, Portland, OR 97207, USA; ehulen@pdx.edu; 6 Health Sciences, Northern Arizona University, Flagstaff, AZ 86011, USA; priscilla.sanderson@nau.edu (P.R.S.); cruz.begay@gmail.com (R.C.B.); 7 Department of Politics and International Affairs, Northern Arizona University, Flagstaff, AZ 86011, USA; candi.corrales@nau.edu; 8 Coconino County, Flagstaff, AZ 86011, USA; jayesplain@gmail.com

**Keywords:** community engagement, evaluation, capacity building, resilience, campus community partnerships

## Abstract

In 2020, global injustice has taken center stage during the uprising of the Black Lives Matter movement and other social movements. Activists are calling attention to longstanding disparities in health outcomes and an urgent need for justice. Given the global socio-political moment, how can health researchers draw on current critical theory and social movements to create structures for equitable outcomes in health research and practice? Here, we demonstrate principles for effective health research and social justice work that builds on community-engaged approaches by weaving critical Indigenous approaches into structural project designs. Our project, “Health Resilience among American Indians in Arizona”, brought new and seasoned researchers together to collect and analyze data on the knowledge of healthcare providers concerning American Indian health and well-being. Four years after the conclusion of the project, the team developed and created a post-project self-assessment to investigate lasting impacts of project participation. In this communication, we discuss the principles of defining and measuring the capacity to build together. This work responds to the call from Indigenous scholars and community leaders to build an internal narrative of change. While we will not present the full instrument, we will discuss building a strong foundation using the principles of engagement for planning and implementing justice and change.

## 1. Introduction

The complexity of global and local crises is immense. There is not a single solution that can solve urgent, health-related problems. The 2020 uprising of social movements like Black Lives Matter, which began after Minneapolis police officer Derek Chauvin murdered George Floyd, brings to light many years of longstanding inequalities in life and health. Indigenous peoples have also faced health disparities and threats to their well-being and life at higher rates than their nonindigenous counterparts. The relationships between social justice and health are not new for people who live with these threats, nor are relationships that have been unexplored by social scientists and public health researchers.

Public health researchers and social scientists have turned to community-engaged approaches to address unequal health outcomes. These approaches provide well documented success in advancing health through multiple forms of knowledge [1,2]. Engaged approaches are strongest when they incorporate methods for collapsing divides between communities and institutions, specifically when including people who have more or less economic and political power. Indigenous leaders suggest a need for research designs that are developed “with” instead of “on” people in ways that provide opportunities for “counter-storytelling” [3]. It is in this confluence of approaches that new narratives of impact emerge. This communication presents the principles behind the development of an internal measure of capacity building to discuss a foundation for justice in engaged research that can contribute to narratives of change.

### 1.1. Community-Engaged and Indigenous Approaches

Community-based participatory research (CBPR) and other engaged approaches share goals of collapsing divides between researchers and the researched by equally incorporating different skills and forms of knowledge. These approaches differ from those that exclusively center professional research skills [4,5,6,7]. However, underlying principles of engagement, do not always result in equitable outcomes of project research and practice. Even when working toward justice, benefits of health-focused projects are sometimes unequally weighted. For example, people who already have leadership positions in hospitals, health clinics, schools, and academic researchers, may obtain more measurable benefits from grant-funded projects than community researchers who bring valuable insider knowledge and skills [8,9]. For instance, the evaluation of a project may only measure the impact on people who are considered to be “beneficiaries” instead of collaborators. A more inclusive look at capacity might reveal that tenure-track university researchers have gained additional grant experience, which is advanced in scholarships, promotions, publications, and other benefits. The short-sightedness of measuring only one type of capacity building leaves equity in benefits from funding unexplored. Developing a more inclusive narrative of how all partners benefit from the project allows groups to understand if benefits are equally weighted [10]. One way of capturing a more inclusive look at how multiple communities benefit from engagement is to involve all project partners in the evaluative process that incorporates multiple perspectives and reflects on the long-term impact that aligns with and draws on Indigenous approaches to research [11,12]. This shift also places trust in the building and the skill development at the center of project goals rather than considering them to be a side effect of partnership projects.

Our goal in developing a collaborative process for capacity building was in part to create and understand more fully what capacity building looked like for all members of the team instead of focusing only on members of a “community” outside of leadership. We defined the layered definition of capacity building that our group developed together in the results section. The authors of this article include members of original research and post-project evaluation teams. Here, we share principles and strategies to continue the conversation of how to measure and interpret capacity building through a process of narrative creation and expand our discussion of the principles used to create a process fully engaged with a research team and community-based and Indigenous theories of change.

### 1.2. Context

The parent project for the development of the evaluation was called “Health Resilience among American Indians in Arizona”, (hereafter “Health Resilience”), which is funded by the National Institutes of Health (NIH) and National Institute on Minority Health and Health Disparities (NIMHD), under the Center for American Indian Resilience (CAIR). “Health Resilience” built on prior engaged studies to investigate health and patient–provider perceptions. Project leads (a health scholar and medical anthropologist) developed a strategy to identify and hire community members to join the team as researchers. Past employment and degree status were not considered in the hiring process intentionally to reflect a community-engaged strategy [13]. Community researchers became paid employees beginning with a multiday intensive training session and continuing into data collection, analysis, implementation, and dissemination. The “Health Resilience” project team included community and academic researchers of different tribal affiliations, ages, genders, and life experiences. Data collection included semi-structured interviews, focus groups, and Wellness Mapping activities [14]. Researchers obtained permissions through the American Indian led local organizations and a university institutional review board (IRB). The team gained permission from the Navajo Nation Human Research and Review Board, Hopi Tribe, and Indian Health Service. Once the multiyear project concluded, the team analyzed data and wrote about, presented, and continued “Health Resilience” in different ways.

### 1.3. Building a New Instrument Together

Researchers built capacity by creating their own instrument based on experiences with the project and questions they had about lasting change after the conclusion of “Health Resilience”. This emergent model reflected a process similar to “counter storytelling” [3] as an epistemological approach to building knowledge. Indigenous researchers have discussed how to make research and evaluation “culturally responsive”, being called culturally responsive indigenous evaluation (CRIE) [15]. This varies from a research strategy that uses a vetted evaluation instrument in a local site. In the case of “Health Resilience”, we specifically created the process to define and understand capacity and relationship building while developing an internal narrative. We did not use a CRIE-specific research design, though we did develop our instrument through a similarly emergent process that focused on capacity and relationship building, which is in line with Indigenous approaches to research.

Understanding why the group chose to develop a narrative requires replacing a traditional research focus with one that is steeped in community engagement and the values of Indigenous focused research. Findings from a recent systematic review found that CBPR projects in Indigenous contexts yielded improved research and capacity and, at the same time, there was a need for projects that improved rigor by defining research questions in partnership [16]. Over half of the projects reviewed relied exclusively on researcher-defined questions that had not been developed with community member involvement. Research with Indigenous peoples has been identified as an area where existing evaluation tools are lacking, and where there are opportunities for using principles of engagement to create a new narrative rather than relying on an existing one [12]. Community-, culture-, and language-focused programs tend to show improvements in health, relationship building, and sustainability of change [17]. In one example, a community-engaged approach was used to develop a “healing model of care” for Indigenous people by building crucial partnerships [18]. These partnerships led to the development of dynamic partnerships that contributed methodologically and in practice to evidence-based models of care. In another example, a research partnership with a Native American community developed procedures for measuring CBPR, which included a focus on measuring “level of participant involvement” and “community voice” to be used in the measure trust and “trustworthiness” of an engaged and participatory project [5]. Indigenous researchers highlight the importance of building a narrative for evaluation together [5]. The development of the assessment allowed for an organic structure of openness to emergent narratives of change [19,20]. The team positioned “community” as a series of overlapping groups including academic researchers and highlighted the need to “integrate knowledge and action for all partners”, as suggested for use of community engagement in tribal contexts [21]. In our team, there was no definitive line between community and academic partners in that some community members had or have since obtained positions affiliated with the only university in the region, while others had full time appointments there. Over half of the researchers were Indigenous or American Indian with different tribal affiliations. This framework of a broad understanding of community beyond academia and non-academia allowed team members to re-think how people work together to challenge and shift power structures in campus–community partnerships.

### 1.4. Defining “Communities” in Engaged Research

A collaboration that crosses institutional, tribal, social, and other boundaries relies on trust, transparency, and tangible reciprocity to function well. Achieving these principles requires project designs and measures that include different communities as both targets of health programming and recipients of the benefits of community-engaged work [3,22]. Community-engaged and Indigenous-focused approaches to research attempt to decolonize research and methods by reducing the traditional categories of researchers and the researched and positioning people who may not have academic degrees as “cultural experts” over their neighborhoods and social spaces [23,24,25]. These approaches recognize the benefits of including people from outside institutional settings. For instance, noninstitutional team members learn new skills like team-building and institutional team members learn how to collaborate and learn from the people in the communities of study.

In limiting a definition of “community” to people outside of academia and clinical settings, researchers can fail to incorporate a full understanding of how researchers themselves constitute a community, or series of overlapping communities. Project leadership at health clinics, hospitals, universities, or other entities constitute a set of communities in the same way that neighborhoods encompass a set of overlapping communities. Community-engaged research offers an opportunity to examine benefits and capacity changes over time in multiple communities, including those who already occupy positions of power. Ongoing collaborative evaluation helps to ensure that outcomes are reaching through barriers to support equity where it is needed most. The development of evaluation in this setting rests on current challenges to empirical models of data collection and analysis discussed by Indigenous researchers and by drawing on foundations that shift the researcher–researched roles to develop new narratives [24,26]. Decolonizing implicit bias of power in definitions of “community” in research is necessary for the health and justice of those communities involved in research activities [26].

### 1.5. Project Assessment in Relation to Parent Project

Methods for the parent project were designed collaboratively with a group of researchers. The parent project grew out of a series of community-engaged projects. Those prior projects resulted in questions about patient–provider communication and American Indian resilience. Upon funding, project leads developed a hiring strategy to recruit and select researchers from communities of study who were skilled at listening and critical thinking regardless of education or work experience. Once the team came together, there was intensive multiday, collaborative training. Intensive research for the parent project lasted 6–8 weeks, with additional weeks for transcriptions and ongoing analysis. This post-project activity happened long after the conclusion of the parent project.

At the conclusion of the project, members of the team continued to stay in contact and work with one another in different capacities. We were curious about how the project impacted all of us, and wanted to ask ourselves and each other about impacts that stayed with us. Evaluation models that exist help groups to explore and understand community coalition functioning and capacity building [10,27]. In addition, the creation of the process presented an opportunity for group members to continue their work together in the development of an instrument and the dissemination of results. Engaged project approaches and designs include ongoing, iterative evaluation embedded within processes of research and implementation [10]. Development of evaluation was part of the primary collaborative training that the team led and participated in together. Leadership was provided by one of the project principal investigators (PIs). This PI is not an American Indian person, although the other two PIs are. She designed and developed the project based on learnings from Indigenous scholars and practitioners, and on experience from developing best practices for community engagement with partners outside of academia. This detailed description of the process of development and evaluation occurred on the foundation of a project structure in a communicative environment.

1. During research and analysis, team members discussed doing a post-project evaluation together. No one person prioritized this process. Instead, it was part of the ongoing discussions during analysis meetings.
2. After the project ended, several members of the group stayed in touch through professional and friendship connections. One project lead and the person who had served as a research specialist discussed checking in with the group to determine the remaining interest in evaluating the long-term impacts of the project.
3. A project lead had sent an inquiry via email to the team. All but one team member responded that they would be interested in participating. The team was unable to locate the remaining member of the team. Everyone was invited to participate in the discussion, which was possible because of trust established during the parent project.
4. The group circulated an inquiry asking people to create questions they would like to have answered about lasting impacts of the project and post-project reflections. All team members responded to the email with ideas.
5. An outside researcher organized the questions into an online survey and analyzed results. This decision was intentional so that the organization of the questions would occur by someone who was not involved in the original project, and therefore did not have a heavy investment in project outcomes. Only the outside researcher had access to the raw data to analyze results and to share the results with team members.
6. All members of the team with the exclusion of two people, who were not available, participated in the development and writing of the manuscript.

## 2. Materials and Methods

Methods specific to the assessment include project partners reconnecting to create an evaluation instrument to assess capacity at an individual level and to evaluate lasting impacts. The project fostered ongoing engagement and capacity building for team members outside of and within university settings. These relationships provide opportunities for employment, raises or promotion, entrance into graduate programs, new partnerships, new funding proposals, and other tangible opportunities. Working together on equal ground to develop the evaluation instrument demonstrated our ability to work together professionally in the years after the completion of the research. Final questions and answers reflected our ability to quickly pick up where we left off at the end of the project, and reflected use of our relationships and skills to complete the evaluation. This process was evaluative in practice and in outcomes.

## 3. Results

The most salient outcome of this process was the development of our own way of thinking through and defining lasting capacity building. Capacity building has been defined elsewhere as community-level outcomes resulting from leveraging a community’s individual and organizational resources toward addressing collective problems and resources, relationships, and leadership [28]. Approaches to community engagement can add skills, knowledge, experiences, new partnerships, and a breadth of experience to deepen individual and community level knowledge and experience. Once a community builds on existing skills to increase capacity, there can be many outcomes that go beyond a project and transform into new partnerships, projects, grants, relationships, and wellness [29]. We drew on these definitions to develop an operationalized concept of capacity building that reflects increases in skills, knowledge, and ability to design and perform related projects; new and/or improved skills that lead to different employment and educational opportunities; new generative relationships of trust; and new knowledge that leads to other outcomes beyond the stated project goals. Capacity building includes human relations skills within a collaborative working team, recognition of project timeline and priorities, and a respectful communication style.

Our view of capacity building began with the understanding that it can impact people on an individual level (e.g., a person obtaining a higher paying job) as well as on a community level (a community leveraging partnerships to bring resources to a neighborhood), and even a larger, policy level (new partnerships that lead to local, state, and federal policy change). Capacity building through engagement can add to a skill set and opportunity landscape for community members who may not hold positions of power or have degrees, as well as people who already hold leadership and research positions, by providing a learning experience that leads to better and more holistic relationships between individuals and organizations as the trust and experience grows.

Our perspective on capacity building reaches beyond the idea that capacity is owned, and distributed by people in positions of power. Certainly, people in positions of power can provide training opportunities and access to resources; however, equally important is the ability for project leads and researchers to learn how to engage with communities outside of these entities to challenge inequality. In light of the focus on power and position in engaged work, scholars and partners critique the public health programming that begins with an assumption that researchers hold knowledge and community partners can (and should) learn from them [30,31]. A layered read of capacity building assumes differently positioned groups can learn from one another for a common goal. Researchers and healthcare providers can learn from communities, just as community groups may learn from investigators [30].

## 4. Discussion

In the burgeoning area of community-engaged work, project teams develop plans based on collaborations between researchers and the researched; however, the relationships between researchers and community partners are not always central to the functioning of these projects. There are numerous reasons for the breakdown of equitable and productive relationships between individuals who already have access to resources before a project begins, including policies that result in unequal pay, structures that only involve community members as recruiters, and other system level issues. Indeed, in our own work, we have witnessed projects where inequality between researchers is borne out through unequal employment advancement, lack of transparency in authorship roles in publications, and the weight of ongoing project success falling on people who are already in positions of power. Partnerships and relationships between people working together in a community-engaged project are central to the ability of projects to positively impact health and well-being [29,32].

Scholars and researchers who work in health knowledge and implementation must consider how engaged practice impacts project team members and communities in multiple ways, and how benefits of research and practice are distributed. Highlighting capacity building as a focus of research and practice is one way to measure and understand what works, and to identify possible improvements [30]. This is a multifaceted alternative to more popular approaches that assess programs, typically dealing with a hierarchical set of dichotomies such as patient/provider or client/provider, and cogently answers many critical concerns coming from Indigenous researchers [15,24,26].

In our engaged assessment, we implemented a communal-reflective contingent of community building [24]. The inclusion of multiple team members demystified the barriers usually constructed between academic cultures, students, and community members and opened a space for exploring intersectionality and challenges assumed boundaries of power. This established a balance between flexibility and commitment through collaboration efforts bridging professional and social gaps among different community spheres of influence, which brought about a sense of equitable distribution of knowledge and power. Team members developed relationships that continue on through collaborative work. These relationships reflect a greater ability to engage in ongoing work based on the relational aspects of the research, which are the qualities that Indigenous researchers define as key principles in understanding and conducting research [31,32].

## 5. Conclusions

We recommend that projects with the goal of engaging multiple communities reducing unexplored boundaries of power create a group process of instrument development and analysis. This activity could be implemented annually for multiyear projects and again after the conclusion of the project to explore and measure who benefits from research. It would enable groups to adjust structures to meet the needs of different and overlapping communities participating in research and practice and help to integrate a framework into practice to erode an assumed barrier between a community of need and others. This would be a summative process that could cause community engagement practices to become more deeply woven into present and future project benefits and keep partnerships accountable for areas where benefits could be equitably distributed.
